# High flow nasal cannula improves lung aeration and enhances CO_2_ removal in hypoxemic critically ill patients

**DOI:** 10.1186/2197-425X-3-S1-A176

**Published:** 2015-10-01

**Authors:** T Mauri, N Eronia, C Turrini, G Grasselli, G Bellani, A Pesenti

**Affiliations:** IRCCS “Ca' Granda Foundation, Maggiore Policlinico Hospital, Milan, Italy; University of Milan-Bicocca, San Gerardo Hospital, Monza, Italy; University of Ferrara, Sant'Anna Hospital, Ferrara, Italy

## Introduction

High-flow nasal cannula (HFNC) is a non-invasive respiratory support increasingly applied to hypoxemic acute respiratory failure patients. HFNC decreases dyspnea, improves oxygenation and enhances patient's comfort.

## Objectives

In the present study, to identify the mechanisms underlying the clinical benefits associated with HFNC, we measured in hypoxemic non-intubated critically ill patients the effects of HFNC on lung aeration, patient's effort and CO_2_ washout efficiency.

## Methods

We performed a prospective randomized cross-over study on hypoxemic (PaO_2_/FiO_2_ ≤300 mmHg) non-intubated patients admitted to Intensive Care Unit of San Gerardo Hospital. We delivered the same air/oxygen mix by HFNC (40 L/min) and facial mask for 20 minutes by random order. Continuous recordings of lung aeration and minute ventilation by Electrical Impedance Tomography (EIT) and of inspiratory effort by esophageal pressure swings (ΔPes) were obtained.

## Results

**Figure 1:**
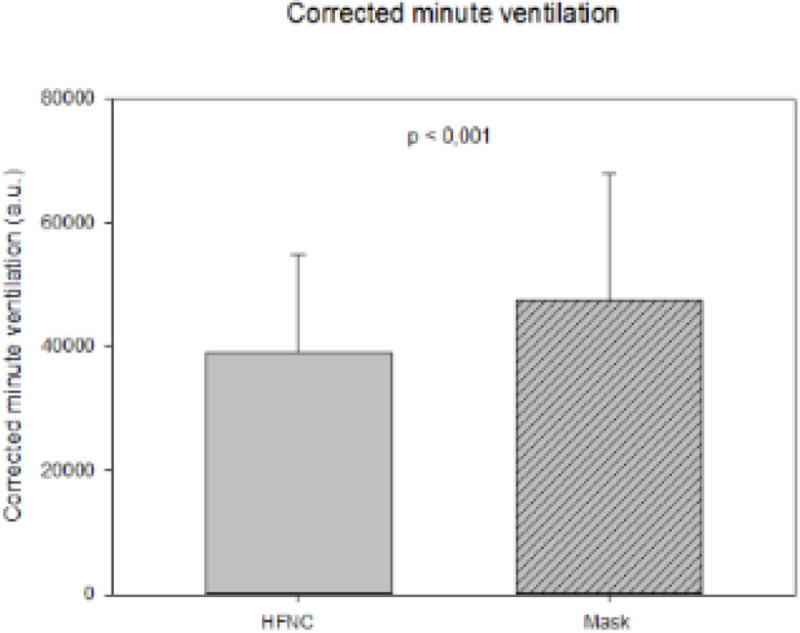
**Corrected minute ventilation.**

We enrolled 15 patients (10 male), aged 57 ± 16 years old. Compared to standard facial mask, HFNC significantly improved PaO_2_/FiO_2_ values (184 ± 53 vs. 130 ± 34 mmHg, p < 0.001). End-expiratory lung volume (867 ± 488 a.u. vs. baseline, p < 0.001) and central venous pressure (6 ± 4 vs. 4 ± 4 mmHg, p < 0.01) increased, too, possibly indicating positive end-expiratory pressure effect. HFNC decreased respiratory rate (22 ± 5 vs. 24 ± 4 bpm, p < 0.001), as well as ΔPes (8.7 ± 3.3 vs. 10.4 ± 3.7 cmH_2_O, p < 0.01). Finally, corrected minute ventilation (i.e., MV*PaCO_2_/40 mmHg, with lower values indicating increased efficiency of CO_2_ removal) decreased (p < 0.001) (Figure 1).

Patients with baseline ΔPes higher than median value (i.e., >8.4 cmH_2_O, indicating higher inspiratory effort) reduced, by application of HFNC, both respiratory rate (-4 ± 3 vs. -1 ± 1 bpm, p = 0.02) and ΔPes swings (-24 ± 17% vs. -5 ± 16%, p < 0.05) most consistently in comparison to patients with lower effort, while oxygenation improved less (+37 ± 22 vs. +79 ± 44 mmHg, p = 0.03).

## Conclusions

In hypoxemic critically ill patients, HFNC might improve oxygenation and patient's comfort by lung expansion and enhanced CO_2_ clearance. Patients with higher inspiratory effort might experience the larger benefit in terms of decreased effort, but higher flows might be needed to optimize oxygenation.

## Grant Acknowledgement

Institutional; Fisher & Paykel Healthcare provided unrestricted research grant to support, in part, the study.

